# Comparison between Exercise Program–Foot Orthoses Treatment and Exercise Program Alone after Pilon Fracture Surgery: Study Protocol for a Randomized Controlled Trial

**DOI:** 10.3390/life13112187

**Published:** 2023-11-10

**Authors:** Andrei-Daniel Bolovan, Roxana-Ramona Onofrei, Gheorghe-Bogdan Hogea, Ahmed Abu-Awwad, Emil-Adrian Lazarescu, Simona-Alina Abu-Awwad, Alexandra-Roxana Tapardea, Madalina-Ianca Suba, Elena-Constanta Amaricai

**Affiliations:** 1 Doctoral School, “Victor Babes” University of Medicine and Pharmacy, 300041 Timisoara, Romania; andrei.bolovan@umft.ro (A.-D.B.); alina.abuawwad@umft.ro (S.-A.A.-A.); tapardea_roxana@yahoo.com (A.-R.T.); madalina.suba@umft.ro (M.-I.S.); 2 “Pius Brinzeu” Emergency Clinical County Hospital, Bld Liviu Rebreanu, No. 156, 300723 Timisoara, Romania; hogea.bogdan@umft.ro (G.-B.H.); lazarescu.adrian@umft.ro (E.-A.L.); 3 Department of Rehabilitation, Physical Medicine and Rheumatology, Research Center for Assessment of Human Motion, Functionality and Disability, “Victor Babes” University of Medicine and Pharmacy, 300041 Timisoara, Romania; onofrei.roxana@umft.ro (R.-R.O.); amaricai.elena@umft.ro (E.-C.A.); 4 Department XV—Discipline of Orthopedics—Traumatology, “Victor Babes” University of Medicine and Pharmacy, Eftimie Murgu Square, No. 2, 300041 Timisoara, Romania; 5 Research Center University Professor Doctor Teodor Șora, “Victor Babes” University of Medicine and Pharmacy, Eftimie Murgu Square, No. 2, 300041 Timisoara, Romania; 6 Department of Anatomy and Embryology, “Victor Babes” University of Medicine and Pharmacy, 300041 Timisoara, Romania; 7 Department XII—Discipline of Obstetrics and Gynecology, “Victor Babes” University of Medicine and Pharmacy, Eftimie Murgu Square, No. 2, 300041 Timisoara, Romania

**Keywords:** gait analysis, myotonometry, pilon fracture, foot orthoses, balance, muscle strength

## Abstract

The management of tibial pilon fractures is challenging and often leads to complications and limitations in ankle function. The study aims to investigate myotonometric parameters and muscle strength of ankle muscles, as well as gait pattern and balance among patients following surgical treatment of pilon fractures. The randomized controlled study will analyze the differences between the patients who will follow a 3-month physical exercise program and will wear customized foot orthoses (i.e., customized orthotic arch support insoles) versus patients who will attend only the physical exercise program. For each group, at least 21 patients will be required. The assessment involves four different testing procedures: myotonometry (anterior tibialis, medial and lateral gastrocnemius, and longus peroneus assessed using MyotonPRO), muscle strength testing (ankle dorsiflexors, plantar flexors, and peroneal muscles assessed using MicroFET2 dynamometer), analysis of gait parameters (using Scheinworks treadmill), and double-leg and single-leg balance tests (using K-Force plate). After 3 months, the assessments will record which of the two treatments (physical exercise program with or without wearing customized foot orthoses) has better outcomes in regaining ankle muscle properties and tone, as well as the restoration of gait and balance.

## 1. Introduction

Pilon fractures are not very common, leading to increased rates of complications and poor clinical outcomes especially in terms of walking impairments [1,2,3]. Various methods of stabilizing pilon fractures are used in clinical practice. Treatment usually consists of open reduction and internal fixation or limited internal fixation combined with an external fixator. These types of fractures are mostly caused by high-energy trauma; they are frequently associated with soft tissue injuries requiring repeated surgery for definitive management and generating thus additional costs, as well as long recovery periods [2,4]. In many cases, the functionality of the affected ankle or leg is not fully regained, severely affecting lifestyle among those affected [5,6,7,8]. There are various publications in the literature that discuss treatment techniques for pilon fractures and evaluate their clinical, functional, and radiological outcomes [1,2,3,4,5]. After sustaining a tibial pilon fracture, individuals may experience long-term complications that impact their quality of life. This is especially true for comminuted intra-articular fractures caused by high-energy trauma. Studies have shown that patients with tibial pilon fractures have lower scores on health-related quality of life questionnaires than uninjured age-matched populations and even people with chronic diseases like AIDS, diabetes, or asthma [5,6]. Patients who underwent surgery for this type of fracture also report a notable loss of ankle joint function and the presence of daily pain. Returning to work may be difficult for these patients, with only 57% of them returning to professional activities after 12 months [5]. Additionally, posttraumatic arthrosis is a common complication of tibial pilon fractures. Studies have revealed that clinical outcomes can worsen with the passage of time. In a study conducted by Chen et al. [9], patients who have undergone open reduction and internal fixation treatment for severe tibial pilon fractures experienced a significantly higher incidence of posttraumatic arthrosis after 2 years, which further increased at the final evaluation (averaging 10 years). Patients with more severe soft tissue injuries at the time of presentation had poorer clinical outcomes [7]. Moreover, the adequacy of reduction was found to be a predictor for long-term outcomes [5].

Gait parameter analysis is a commonly used method to evaluate the functional performance in various populations [10,11,12]. However, only a few studies have analyzed gait parameters after tibial pilon fractures, which are crucial indicators of the effectiveness of the surgical treatment [13,14,15,16]. When compared to healthy controls, patients with pilon fracture who needed surgery and immobilization present altered gait patterns and clinical symptoms [13,14]. Similar to other studies [7,14], in our study, the non-injured ankle on the opposite side will serve as the healthy control. Soft tissue injuries, prolonged immobilization, and extended periods of non-weight-bearing activity on the injured side resulting from tibial pilon fractures can lead to muscle atrophy, decreased strength, and stiffness [17,18]. Pilon fractures can also lead to balance disturbances [19]. The altered balance can greatly impact walking and functional mobility, thus limiting daily activities and reducing the overall quality of life [19,20,21]. Better understanding of the gait parameters and leg muscle properties alteration after pilon fracture surgery could improve therapeutic procedures. It is important to note that successful treatment should result in improved joint mobility, restored muscle strength, and reduced pain and swelling, that ultimately lead to normalized gait parameters and optimal functioning in everyday and sport recreational activities [12,22,23,24,25,26,27]. According to a systematic review, people who performed active exercises after ankle surgery were able to return earlier to daily activities and work [28]. The use of orthotics is a frequently used practice for various neurological and musculoskeletal conditions for improvement in functional mobility and increased quality of life [15,29,30]. The foot orthoses are used to regain walking ability, correct foot deformities, and maintain the stability of lower limb joints. Customized orthotic design promotes patient rehabilitation, especially in restoring natural gait patterns [31]. To our knowledge, analysis of gait parameters, balance, and muscle assessment before and after rehabilitation has not yet been performed among patients with surgically treated pilon fractures. In our study, we hypothesize that patients with unilateral pilon fractures will have improved gait parameters, balance, muscle strength, and functional parameters after following a 3-month exercise program and wearing foot orthoses. The parameters of the affected ankle should be similar to those of the contralateral non-injured ankle.

### Study Objectives

This study aims to conduct a comprehensive assessment of the muscles and gait parameters in patients who have undergone surgery for pilon fractures and compare the results before and after a physical exercise training program with or without wearing foot orthoses represented by customized orthotic arch support insoles. The purpose of our study is to investigate whether combining foot orthoses with an exercise program leads to improved gait parameters, balance, and ankle muscle properties compared to the exercise program alone.

## 2. Materials and Methods

This protocol was developed in accordance with the SPIRIT guidelines [32] and describes an outcome-assessor-blinded, two-arm, parallel-group, randomized controlled trial. Subjects will be randomly allocated (using a system of random number tables by one investigator who will not be involved in the recruitment process and data collection) into either the group that will wear foot orthoses in addition to physical exercise (Group 1) or the group that will only perform physical exercise training (Group 2). The investigators who are involved in data analysis, baseline, and follow-up assessments will be blinded to group allocation.

### 2.1. Sample Size Calculation

The sample size was calculated using G*Power 3.1.9.7 (Heinrich-Heine-Universität, Düsseldorf, Germany), with a significance level of 0.05, 0.8 power, and an effect size of 0.8. A total of 42 subjects will participate in the study (21 per group) [33].

### 2.2. Recruitment and Informed Consent

This study includes patients who underwent surgical treatment for tibial pilon fractures. Prior to the study, patients will be presented with both verbal and written information regarding the study. They will then be required to provide written consent. The study is conducted in accordance with the Declaration of Helsinki. The study was approved by the Ethics Committee of the Victor Babes University of Medicine and Pharmacy Timisoara (reference no. 26/2023-08-25) and it was registered in the Iranian Registry of Clinical Trials on 28 August 2023 (reference no. IRCT20230813059137N1).

#### 2.2.1. Eligibility Criteria

**Figure 1:**
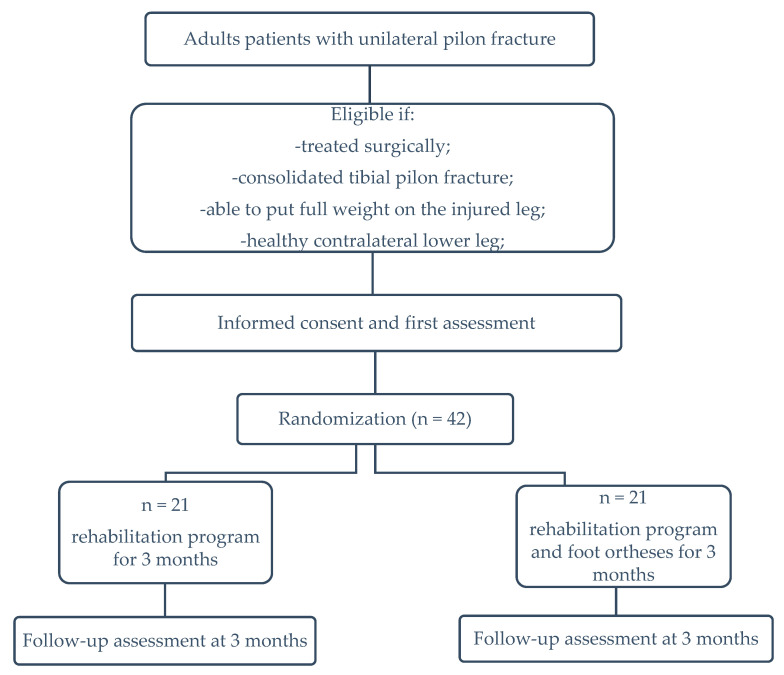
Flow chart of study design.

The following are the requirements for participation in this study: patients must be adults who have undergone surgery for unilateral tibial pilon fracture; they must show clinical and radiological evidence of fracture healing; they must be able to apply full weight bearing onto the affected leg; they must agree to participate voluntarily. In addition, it is necessary for them to have a healthy contralateral lower limb that can be used as a healthy control (Figure 1).

#### 2.2.2. Exclusion Criteria

Individuals will be excluded from the study if they have a history of traumas or fractures in the affected lower limb, a history of traumas or fractures in the opposite lower limb (used as healthy control), any neurological or other health conditions that may cause difficulty in walking or changes in muscle function, or lower leg asymmetry not related to the tibial pilon fracture. Patients with psychiatric disorders or severe cardiovascular disease, morbid obesity (BMI > 40), or cancers will be excluded from the study due to the potential of these conditions to affect compliance and limit the follow-up [34].

#### 2.2.3. Discontinuing Criteria

Any patients who do not use the foot orthosis or fail to comply with the exercise program will be excluded from the study. Also, patients who, during the rehabilitation period, suffer any lower limb injury or any injury that may affect gait or muscle strength will be excluded from the study.

#### 2.2.4. Interventions

Before the baseline assessment, patients will be divided into two groups. Both groups will undergo an identical exercise program. Group 1 will also wear carbon foot orthoses with full reinforcement specially designed to correct the asymmetries detected on the first set of measurements. Gait parameters analysis can help determine specific movement asymmetries [12,13,14]. The Scheinworks treadmill will be used for this purpose, allowing a practical equilateral overview of important gait parameters [35]. During the analysis, the following parameters were taken into consideration: maximum force exerted by the forefoot and backfoot (expressed as a percentage in relation to body weight); step length (measured in centimeters), which is the distance between the foot contacts on opposite sides of the body; stance phase (expressed as a percentage), which is the period of the gait cycle when the foot is in contact with the ground; swing phase (also expressed as a percentage), which is the period of the gait cycle when the foot is not in contact with the ground; step time (measured in seconds), which is the time between the heel contact of one side of the body and the heel contact of the opposite side; stride time (measured in seconds), which is the time taken for the left and right limbs to complete one stride; step cadence which is the number of steps taken per minute, and velocity (measured in kilometers per hour), which is the speed of gait [12,16,35]. A blueprint or scan of the feet will also be performed. Having determined the correct shoe size, a trial insole will be made to fit the diagnosis. Further corrections can be made if the desired gait pattern has not yet been achieved. In most cases, the insole’s influence should be directly recognizable after a couple of steps. The insoles will be designed for both injured and non-injured side based on patients’ specific needs. The movement cycles can be recorded with patients going barefoot, wearing shoes, or foot orthoses [36]. The carbon element that stiffens the orthosis is narrow, while the cover is wide, allowing for optimal fitting in various shoes without causing damage to the carbon [35], so that patients can wear them every day regardless of the type of footwear used and the type of activity undertaken.

### 2.3. Exercise Program

The exercise program will begin in a rehabilitation center and continue as a home exercise program. The first three sessions will be supervised by a physical therapist at the center to ensure that patients perform all exercises correctly and learn how to exercise appropriately at home. The physical therapist will be blind to patients’ baseline measurements.

The entire exercise program will last for 3 months, with a frequency of 5–7 times per week. Each session will last between 30 and 45 min.

The objectives of the exercise program are to increase the range of motion in the affected ankle, strengthen the affected ankle muscles, improve stability in the injured lower limb, coordinate both lower limbs, retrain walking, and regain previous recreational abilities (running, jumping, climbing, cycling). The goal of increasing stability is to prevent ankle sprains, as patients with this condition have a high risk of spraining their affected ankle [12].

The program includes exercises for ankle mobility (dorsiflexion, plantar flexion, inversion, and eversion) and strengthening (concentric contractions of the anterior, posterior, and peroneus muscle groups, thera-band (Figure S1), and light ankle sandbags exercises (Figure S3a)) [37]. Patients will also perform walking forward and sidewards (without obstacles and with obstacles), walking backward, and up and down stairs (Figures S4 and S5). The program will also include the use of a wobble cushion (Figure S3b) and single leg exercises to improve balance and ankle stability (Figures S2 and S3c) [37,38].

Patients’ compliance will be assessed using daily registration in a logbook [39]. It is necessary for the patients to keep track of the number of sessions per week and the duration of each session. Compliance will be calculated by dividing the amounts of exercises actually performed by the prescribed amount of exercise (5–7 sessions of exercises per week, 30 to 45 min for each session). Furthermore, participants will record other medical treatments or injuries that occur during the rehabilitation period in the logbook. If participants have a compliance score of less than 50% (i.e., less than 3 exercise sessions per week), they will be excluded from the study.

### 2.4. Measurements

Dynamic pedography will be used for gait analysis to assess static and dynamic plantar pressure and changes that occur before and after completion of the exercise program and after using the customized insoles. A myotonometer will be used for muscle properties assessment. Muscle strength will be tested with a digital handheld dynamometer. The contralateral non-injured ankle will serve as a control.

#### 2.4.1. Gait Analysis

During gait analysis, the patient will be asked to stand upright on the Scheinworks treadmill (Model FDM-TDSL-3i). Initially, a static measurement of plantar pressure will be taken. Before starting their treadmill exercise, the participants will receive a safety briefing and a 3 min familiarization period for walking. Then, patient will begin walking on the treadmill while dynamic measurements are recorded. The treadmill features a plantar pressure measuring plate integrated underneath it, which has capacitive sensors to measure the distribution of plantar pressure not only during orthostasis but also during walking. The sensor plate integrated into the treadmill offers analysis of the pressure, power, time, and step parameters and evaluation of gait symmetry as standard [12,35,40]. The pressure plate has a sensing area of 94.8 × 40.6 cm and incorporates 5376 capacitive sensors, with a resolution of 1.4 sensors/cm^2^, a measurement area of 1–120 N/cm^2^, and precision of ±5% of the final value. The treadmill has a contact surface of 200 × 92 cm and its belt speed can be adjusted between 0.2 and 24 km/h in 0.1 km/h steps [35]. Before the baseline assessment, the patients will be given a trial using the platform to become familiar with the test method. During this examination, the participants will walk barefoot, while the platform will be calibrated before each attempt. Each patient will be asked to perform five trials, out of which the average of three good attempts for each evaluated parameter will be considered for analysis. A good attempt is defined as both feet making contact with the platform at least three times during walking, eyes remaining open throughout the test, walking without stopping at the participant’s preferred speed, and an absence of excessive trunk rotation [12,40,41].

During these analyses, the data interpretation will occur directly after the measurement. The software will be used to calculate spatial and temporal gait parameters and to analyze the data. The results will then be immediately available in the form of a report.

#### 2.4.2. Myotonometric Evaluation

**Figure 2:**
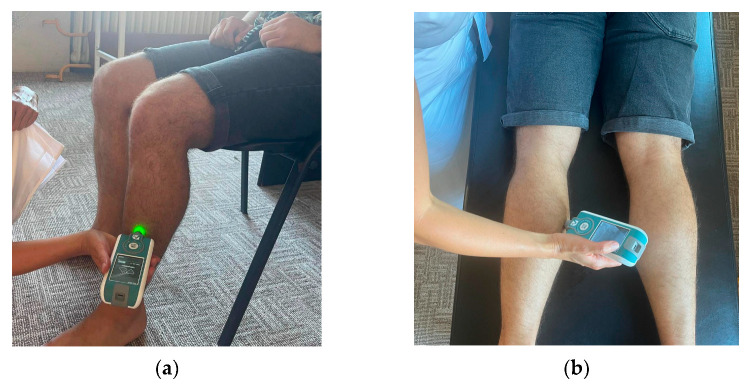
Myotonometric assessment of (**a**) tibialis anterior and (**b**) gastrocnemius muscles using Myoton PRO.

The Myoton PRO Digital Palpation Device with Software v.5.0.0.232 is used for myotonometric assessment [42]. This evaluation takes five minutes per test procedure, which will be conducted in two rounds. The patients will be tested for leg muscles in different compartments while in a supine or seated position for the anterior and lateral compartment and in a relaxed prone position for the posterior compartment. Only the superficial muscles of the leg will be assessed using the myotonometer. The tibialis anterior muscle will be tested in the anterior compartment (Figure 2a). The gastrocnemius muscle will be tested in the posterior compartment of the lower leg (Figure 2b). The peroneus longus muscle will be assessed in the lateral compartment. Similar to previous studies, the medial and lateral gastrocnemius muscles will be measured at one-third distal to the lower leg length in line with the popliteal crease to the malleolus [43], the peroneus longus muscle will be measured at a proximal one-third from the head of the fibula to the lateral malleolus [44]. The tibialis anterior muscle will be measured at one-third of the distance between the tibial tuberosity and the lateral malleolus. The muscle belly (lateral to this line) will be palpated during gentle resisted isometric contraction (ankle dorsiflexion) [45].

The myotonometric assessment will be performed for both the affected and non-affected lower extremities. The device records the natural damped oscillation of the soft tissue as an acceleration signal and calculates it with an external mechanical impulse with low force, rapid release, and constant preload. MyotonPRO technology provides an assessment of muscle tone, elasticity, dynamic stiffness, tension state, relaxation time, and relaxation time strain ratio of the targeted muscle [42,46].

#### 2.4.3. Muscle Strength Evaluation

**Figure 3:**
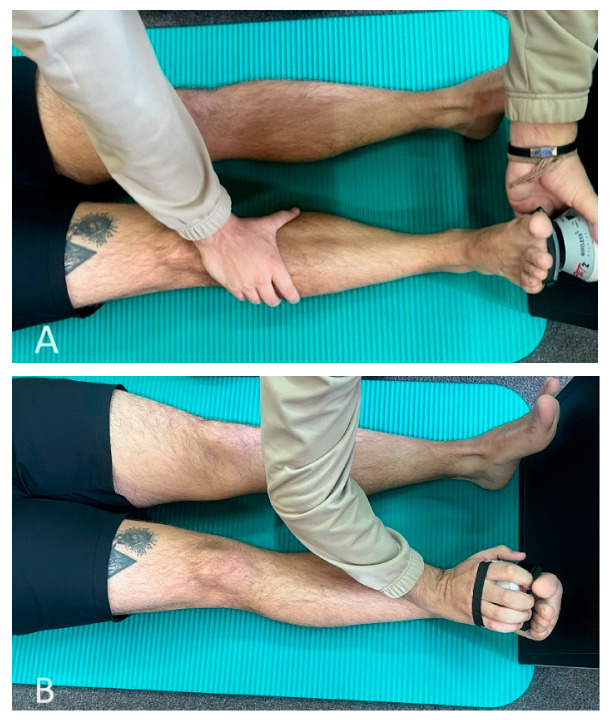
Muscle strength evaluation during (**A**) plantar flexion and (**B**) dorsiflexion using MicroFET2 dynamometer.

For muscle strength testing, a MicroFET2 dynamometer (Hoggan Scientific, Salt Lake City, UT, USA) will be used. The microFET2 Digital Handheld Dynamometer muscle tester is a portable and accurate device that is specifically designed for taking objective, reliable, and quantifiable muscle testing measurements [47]. Hand-held dynamometry is a commonly used method for measuring ankle plantarflexion strength. It has been shown to be a valid, sensitive, and reliable measure of foot and ankle strength. However, the reliability of assessing ankle plantarflexion strength can vary, especially with higher plantarflexion forces [48]. Fixed dynamometers are suggested to improve reliability and are considered the gold standard for measuring both isokinetic and isometric strength [49]. Nonetheless, their high cost and lack of portability can make them less practical for clinical settings. In their study, Davis et al. [48] concluded that both methods of measuring ankle plantar–flexion force are reliable. Hand-held dynamometry alone is more consistent between repeated measures and might be more precise in detecting true change. On the other hand, fixed dynamometers might be more accurate, especially among stronger individuals. In our study, participants will be barefoot and will perform ankle plantar flexion and dorsiflexion while lying supine with their ankles in plantar grade, with their hips and knees extended. The dynamometer will be placed over the metatarsal heads on the sole of the foot for plantar flexion, and on the dorsum for dorsiflexion, and the examiner will apply unmoving resistance during 3 s contractions (Figure 3). For extra stability during ankle plantar flexion assessment, the dynamometer will be positioned against a heavy wooden block. We will repeat both muscle testing procedures for three trials, with a 5 s rest between them. The analysis will consider the mean of the three trials [46]. The muscle strength evaluation will be performed for both affected and non-affected lower extremities.

#### 2.4.4. K-Force Plate

**Figure 4:**
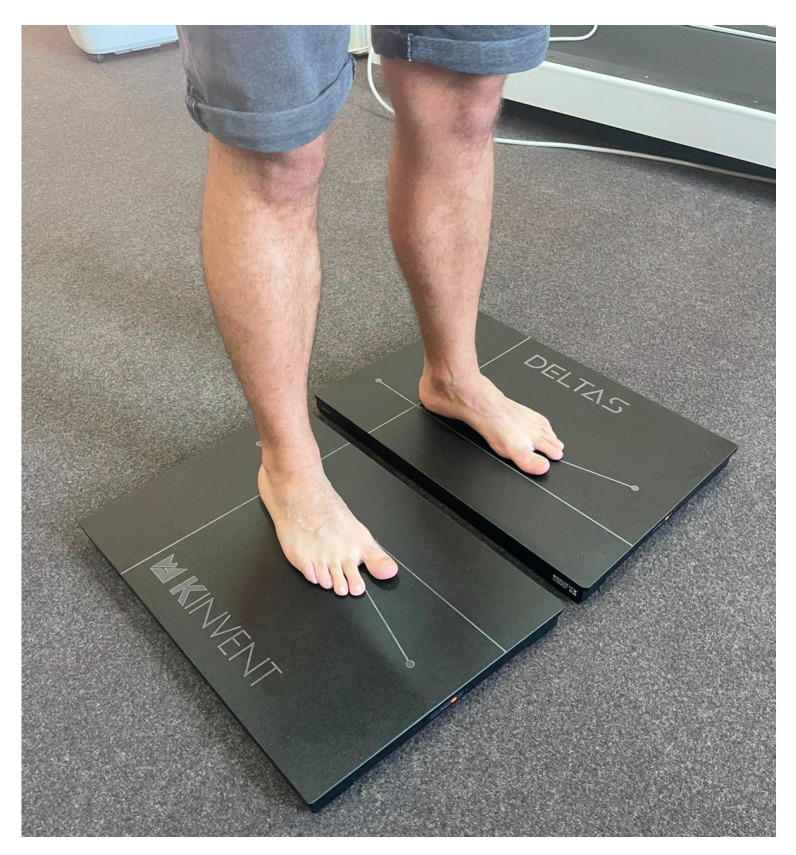
Static balance evaluation using K-force plates.

K-Force plates are a dependable option for examining both static and dynamic balance across a variety of movements such as standing, squatting, and counter-movement jumps [50]. These plates measure both the center of pressure and weight distribution, allowing for an objective assessment of balance (Figure 4) [51,52].

During evaluation, participants will be asked to perform a single-leg balance test under two different conditions. The first condition will involve standing on both the fractured and healthy leg for three repetitions, with open eyes, on the K-Force Plates. The second condition will involve standing on both legs again for three repetitions, this time with eyes closed on the K-Force plates [50,51].

In the single-leg balance test, participants have to stand on one leg for 10 s while focusing on a point 5 m away, with their hands on their hips and the non-load-bearing leg slightly bent at the hip and knee. The test duration of 10 s was chosen based on the time norms of the closed-eye condition during a unipodal balance exercise [51].

## 3. Expected Results

After the first assessment, we anticipate weaker results for the affected leg in terms of myotonometric parameters and muscle strength when compared to the non-affected leg. We believe that the four ankle muscles (anterior tibialis, medial and lateral gastrocnemius, and longus peroneus) on the injured side will display increased frequency and dynamic stiffness, as well as reduced elasticity and a lower relaxation to deformation time ratio. As for isometric muscle force, we expect the ankle dorsiflexors, plantar flexors, and peroneal muscles on the affected limb to exhibit lower values than those of the healthy limb. However, after three months of participating in a physical exercise program, with or without the use of foot orthosis, we predict that there will be no significant differences in myotonometric parameters and muscle strength between the injured and non-injured lower extremity.

Initially, it is likely that there will be a difference in balance while standing on one leg with eyes open between the affected and non-affected limb. However, we anticipate that this discrepancy will decrease significantly within the 3-month exercise program. Additionally, participating in a physical exercise program, with or without a foot orthosis, is expected to improve balance during standing with both conditions of eyes open and eyes closed compared to the first assessment.

At this point, we are not able to make any assumptions about which group—the one using customized foot orthoses and undergoing physical exercise program or the one solely completing the physical exercise program—will have better results regarding myotonometry, muscle strength, and balance.

## 4. Discussion

To date, this will be the first study to assess both the properties of ankle muscles and foot kinematics after tibial pilon fracture surgery. The study will be a randomized one, analyzing the differences between the patients that will follow a 3-month physical exercise program and will also wear customized foot orthoses versus patients that will attend only rehabilitation. The assessment involves four different testing procedures: myotonometry, muscle strength testing, gait analysis, and double-leg and single-leg balance tests.

Myotonometry measures the state of tension (when the muscle is relaxed), biomechanical properties (dynamic stiffness and logarithmic decrement, characterizing elasticity or dissipation of natural oscillation), and viscoelastic properties (mechanical stress relaxation time and the ratio of relaxation time to deformation time, characterizing creep) [42]. Anterior tibialis, medial and lateral gastrocnemius, and longus peroneus will be tested through myotonometry. The anterior tibialis muscle is responsible for ankle dorsiflexion and assists in foot inversion. The longus peroneus muscle’s main action is foot eversion and assists in ankle plantar flexion. Gastrocnemius muscles and the soleus muscle are responsible for ankle plantar flexion. We decided not to test the soleus using myotonometry as it is a more profound muscle [53].

The isometric muscle force of ankle dorsiflexors, plantar flexors, and peroneal muscles will be measured using a hand-held dynamometer [47,48]. We chose to apply dynamometry for these muscles (as a group muscle testing) due to the anatomical and biomechanical features.

The muscles of the lower leg produce different movements in the ankle and foot that are crucial for daily activities such as walking and running. While the individual muscles in each compartment have additional functions, it is important to consider muscle groups as a functional unit. The anterior or dorsiflexor compartment, that contains the tibialis anterior, extensor digitorum longus, fibularis tertius, and extensor hallucis longus mainly produces dorsiflexion of the foot at the ankle joint (an action particularly important for the swing phase of the gait cycle in which the leg is lifted off from the ground). The posterior or plantar flexor group, which consists of a superficial layer comprised of the gastrocnemius, plantaris, and soleus, and a deep layer comprised of tibialis posterior, flexor hallucis longus, popliteus, and flexor digitorum longus muscles, primarily produces plantar flexion of the foot at the ankle joint (an action important for the toe-off phase of the gait cycle, in which the foot prepares to leave the ground). The lateral or fibular group, which consists of fibularis longus and fibularis brevis, mainly produces an eversion of the foot at the subtalar joint. This action plays an important role in maintaining balance while standing on one leg or walking on rough surfaces [53].

Except for the assessment of ankle muscle properties and muscle strength, our study aims to analyze balance and gait. K-Force plates measure the static and dynamic balance in a wide range of conditions (stance, squats) [50]. In the current study, the patients’ balance will be tested during standing (eyes open, eyes closed) and during single-leg standing tests (eyes open). Previous studies have shown that reduced ankle dorsiflexion range of motion is often linked to poor balance and associated activities in ankle fracture patients [19,20]. This connection is supported by research conducted on healthy individuals as well as those with chronic ankle instability, where reduced ankle dorsiflexion range of motion has been found to alter lower limb kinematics and reduce balance, particularly during dynamic actions in the sagittal plane such as single-leg reaching or landing tasks [54].

The use of treadmills with pressure platforms as outcome assessment tools is becoming more common in clinical and research settings. However, there is limited published evidence on the effectiveness of these systems. These treadmills capture electronic footprints which allow for quick measurement of basic gait parameters and the vertical component of ground reaction force over many steps. They have been used to monitor gait patterns in patients with musculoskeletal and neurological disorders [55,56]. Normal gait requires sufficient muscle strength, balance, proprioception, joint mobility, and no pain. Gait analysis is used to measure the effectiveness of a certain therapy by comparing patients’ gait parameters to those of healthy individuals. It enables the assessment of functional outcomes, rehabilitation effectiveness following surgical treatment for musculoskeletal injuries, and individualized treatment and rehabilitation programs [22,23,24,25,26,27]. Restoring gait function comparable to healthy subjects is crucial for patients’ quality of life [19,20,21,57].

Our study will include patients with pilon fractures that have been treated surgically. For the management of these fractures, the most common approach is open reduction and internal fixation with plates and screws. External fixation devices are very useful in fractures with associated soft tissue damage and are used as a temporary reduction and fixation method until osteosynthesis with plates and screws is permitted by the soft tissue appearance or biological status of the patient. External fixation devices can be used in certain cases as a definitive treatment method. A study conducted by Wyrsch et al. [58] compared the effectiveness of open reduction and internal fixation with external fixation as a definitive treatment. Their study revealed that postoperative infections were significantly more common among patients who underwent open reduction and plating. However, there were no statistically significant differences in functional outcomes and complications between the two treatment options. In a meta-analysis by Wang et al. [59], that included nine studies with 498 fractures, no significant differences were found between open reduction and internal fixation, and limited internal fixation combined with external fixation in terms of absence of healing, vicious or delayed healing, superficial and deep infections, arthrosis symptoms, or chronic osteomyelitis.

Tibial pilon fractures often result in complications leading to gait impairment. The lack of improvement in joint mobility results in modified gait parameters. Limited movement in the ankle joint can increase energy expenditure while walking and lead to poorer gait parameters [41]. Pawik et al. [60] observed symmetry in the gait parameters after treating pilon fractures with the Ilizarov external fixator. This method of stabilization allows the restoration of gait parameters with differences from those observed in healthy subjects. In particular, the biomechanics of the lower limbs remain disturbed. The study of Wietecki et al. [16] assessed the kinematic gait parameters in 23 patients with pilon fractures treated with the Ilizarov method. The surgery was performed 24–48 months prior to study measurements. The authors recorded significant limited ankle dorsiflexion, inversion, and abduction of the operated leg in comparison to the non-operated one. They suggested the need for intensive ankle joint rehabilitation following pilon fracture treatment. Houben et al. [13] also stated that patients with pilon fractures showed altered gait compared to healthy controls. There was lower speed and less range of motion between the hindfoot and tibia in the flexion/extension and inversion/eversion, but more range of motion in the abduction/adduction during the push-off phase. The type of fracture was significantly correlated to the passive range of motion.

Patients with tibial pilon fractures usually experience complications in the muscles of the affected limb, leading to gait alteration and difficulties in daily activities. Prolonged rest or immobilization can worsen these complications. Morasiewicz et al. [41] indicated that increased tension in the postural muscles and the higher energy demand related to the shortening and deformation of limbs worsens the parameters of the gait and disturbs its symmetry. The disturbed phase of propulsion may result from the weakening of the gastrocnemius muscle [41]. Aiona et al. [61] reported that shortening of the limbs causes pain and the activation of compensatory mechanisms, which causes higher energy expenditure and affects gait parameters.

To our knowledge, the current study is the first to assess the myotonometric properties of four muscles (anterior tibialis, medial and lateral gastrocnemius, and longus peroneus) in patients after pilon fracture. The study of Halvachizadeh et al. [7] assessed the degree of soft tissue involvement following closed ankle and pilon fractures. Analyzing the mechanical characteristics, the authors noted that the local tension of the soft tissue was affected by the injury; the local frequency and local stiffness were increased, while the stress/relaxation time decreased. In contrast to this study which assessed using myotonometry the soft tissue (soft spot between the lateral malleolus and Achilles tendon), our study will evaluate the muscle myotonometric properties.

Attempting to improve foot and gait biomechanics through the use of orthotics is a commonly used practice for various neurological and musculoskeletal conditions. In the study of Lee et al. [30], they compared the static balance effects of bare foot and ankle–foot orthoses in patients with stroke with foot drop. The measurements showed a significant increase in static balance with the use of orthoses. A study conducted by Quacinella et al. [15] used a dynamic exoskeletal orthosis designed to improve gait for patients experiencing functional loss after a high-energy tibial pilon fracture. The study evaluated various gait parameters such as velocity, cadence, stride length, and single-leg stance. The results showed that only gait velocity improved following the application of the orthosis, while the other gait variables remained unchanged [15,31].

In our study, all patients (group with or without foot orthosis) will attend a 3-month physical exercise program. We anticipate that most of the patients involved will take part in the study between 6 months and 1 year after surgery.

## 5. Conclusions

The randomized controlled study aims to provide options for the long-term management of treatment for patients that have followed surgery for tibial pilon fractures. After 3 months, the assessments will record which of the two therapeutic alternatives (physical exercise program with or without wearing customized foot orthoses) achieves better outcomes in what concern the regain of ankle muscle properties and tone, as well as the restoration of gait and balance.

## Data Availability

The data, representing information about the subjects (baseline, age, gender, height, weight, anamnestic data), as well as the results of the assessments, will be kept and stored within the Department of Rehabilitation, Physical Medicine and Rheumatology of the “Victor Babes” University of Medicine and Pharmacy Timisoara. The results of the study will be disseminated through the publication of articles in peer-reviewed journals, ensuring the confidentiality of the subjects (no publication of subjects’ personal data, photographs or video recordings). Essential documents (subject data, evaluation results) must remain complete and legible throughout the data retention period. The data retention period is 10 years from the end of the study.

## Supplementary Materials

The following supporting information can be downloaded at: https://www.mdpi.com/article/10.3390/life13112187/s1, Figure S1. Subject performing thera-band exercises: (a) ankle dorsiflexion; (b) ankle plantar flexion; (c) ankle eversion; (d) ankle inversion; Figure S2. Subject performing single leg balance with 4 way toe taps; Figure S3. Subject performing (a) light ankle sandbags exercises; (b) maitaining balance on a wobble cushion; (c) single leg plantar flexion; Figure S4. Subject (a) ascending and (b) descending stairs sideways; Figure S5. Subject (a) ascending and (b) descending stairs.
