# Supplementary material for: Comparison between Exercise Program–Foot Orthoses Treatment and Exercise Program Alone after Pilon Fracture Surgery: Study Protocol for a Randomized Controlled Trial
Source: Life (Basel). 2023 Nov 10;13(11):2187. doi: 10.3390/life13112187 (PMC10672240; doi:10.3390/life13112187)
Supplement: Supplementary file 1 [file life-13-02187-s001.zip › life-2600843-supplementary.pdf]

Supplementary material

In this supplementary material we provide a brief visual presentation of some of the exercises that patients will perform at home. The exercise program will begin in a rehabilitation center and continue as a home exercise program.

The entire exercise program will last for 3 months, with a frequency of 5-7 times per week. Each session will last between 30 to 45 minutes.

To improve ankle mobility and strengthening, patients will perform thera-band exercises (i.e. dorsiflexion, plantar flexion, inversion, and eversion)(**Figure S1**)and light ankle sandbags exercises)(**Figure S3a**). Patients will also perform walking forward and side-wards (without obstacles and with obstacles), walking backward, and stairs up and down (**Figure S4, Figure S5**). The program also includes the use of a wobble cushion (**Figure S3b**) and single leg exercises to improve balance and ankle stability (**Figure S2**, **Figure S3c**).

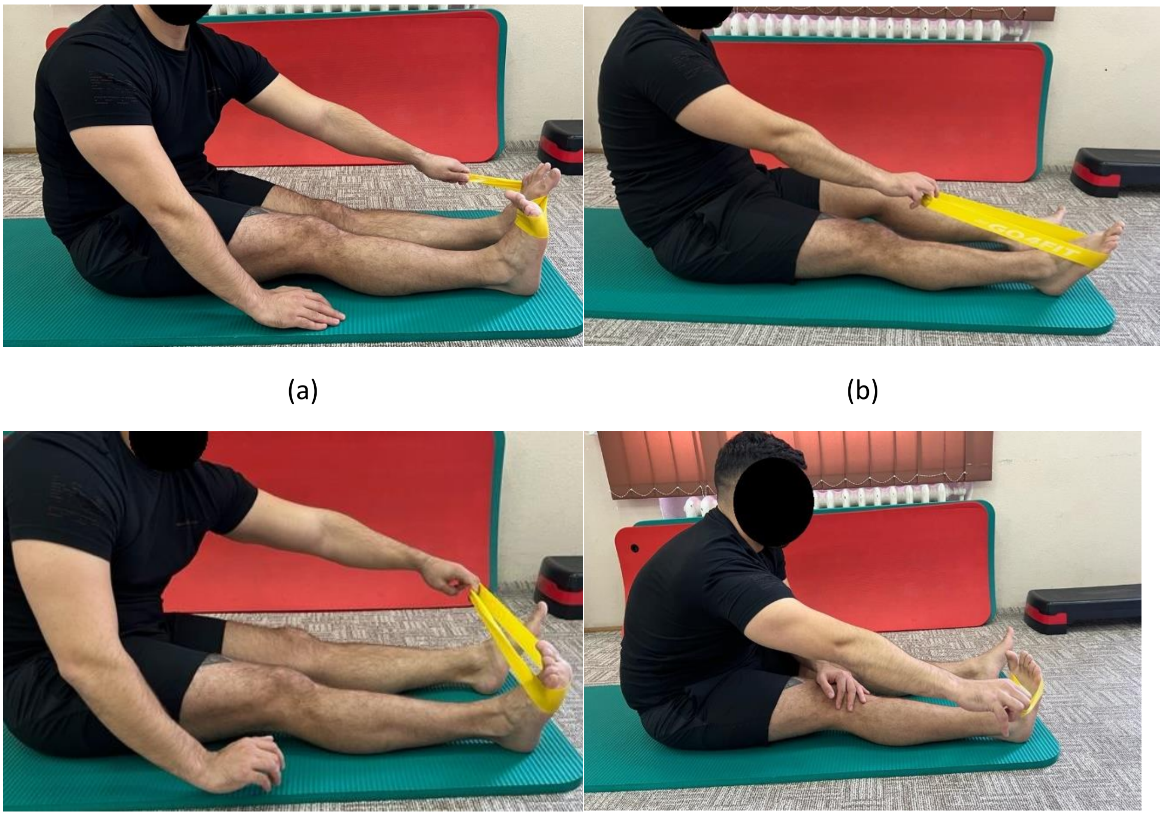

(a) (b)

(c) (d)

Figure S1. Subject performing thera-band exercises: (a) ankle dorsiflexion; (b) ankle plantar flexion; (c) ankle eversion; (d) ankle inversion.

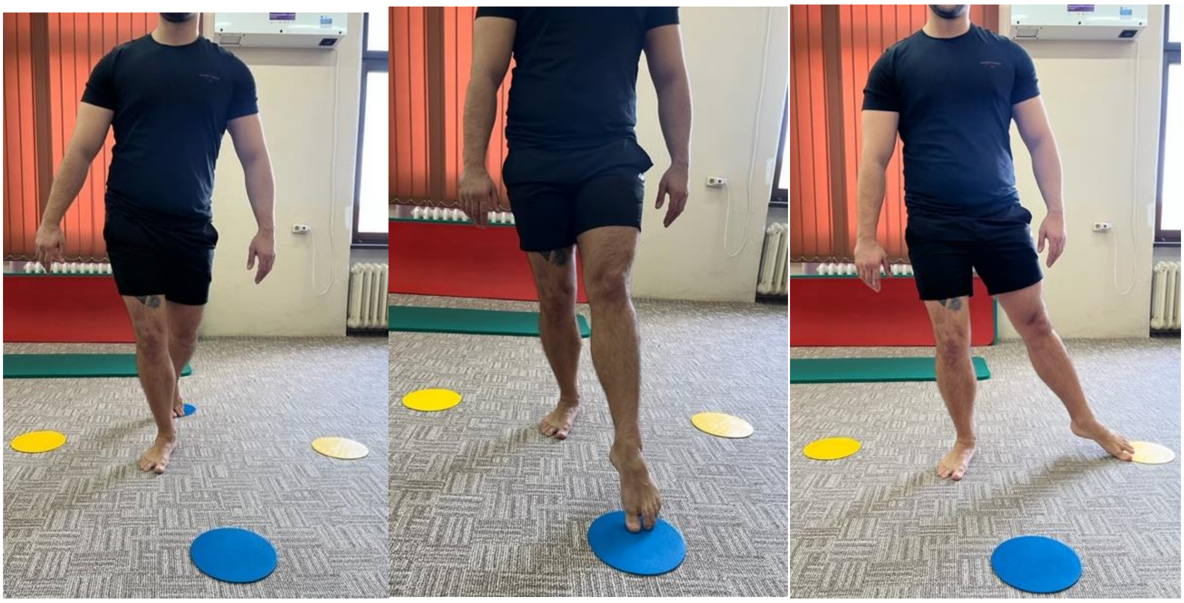

Figure S2. Subject performing single leg balance with 4 way toe taps

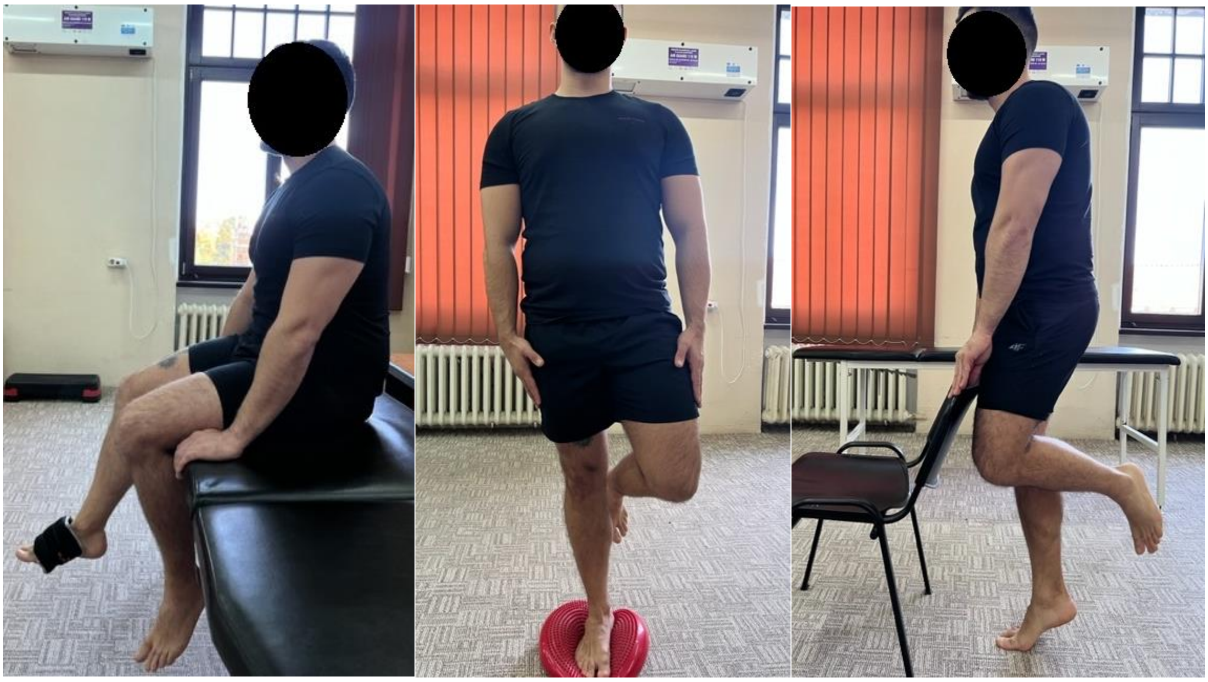

(a) (b) (c)

Figure S3. Subject performing (a) light ankle sandbags exercises; (b) maitaining balance on a wobble cushion; (c) single leg plantar flexion

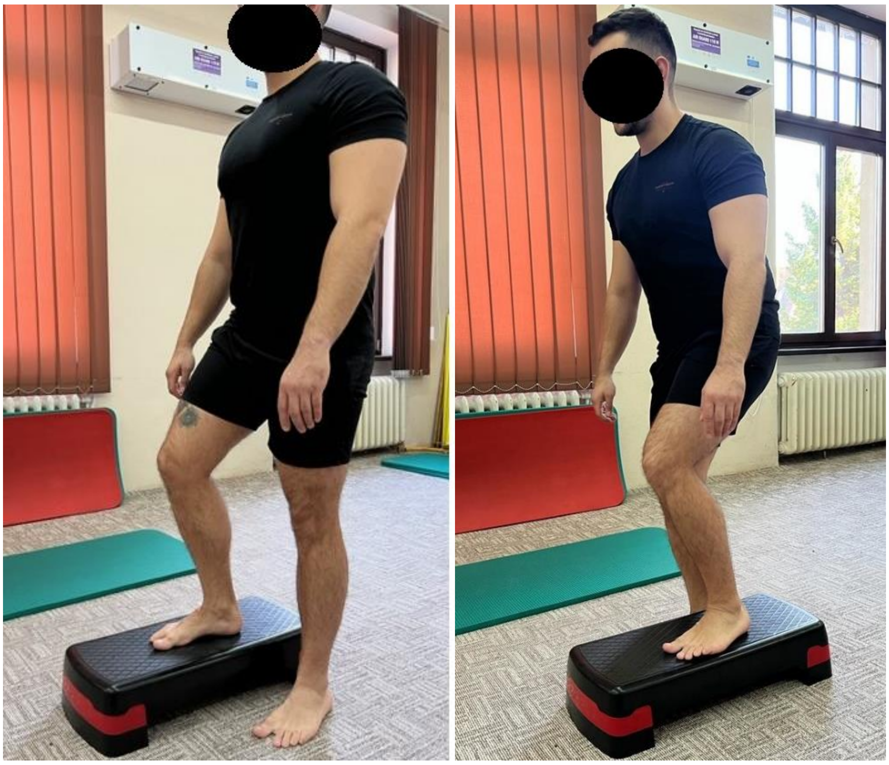

(a) (b)

Figure S4. Subject (a) ascending and (b) descending stairs sideways

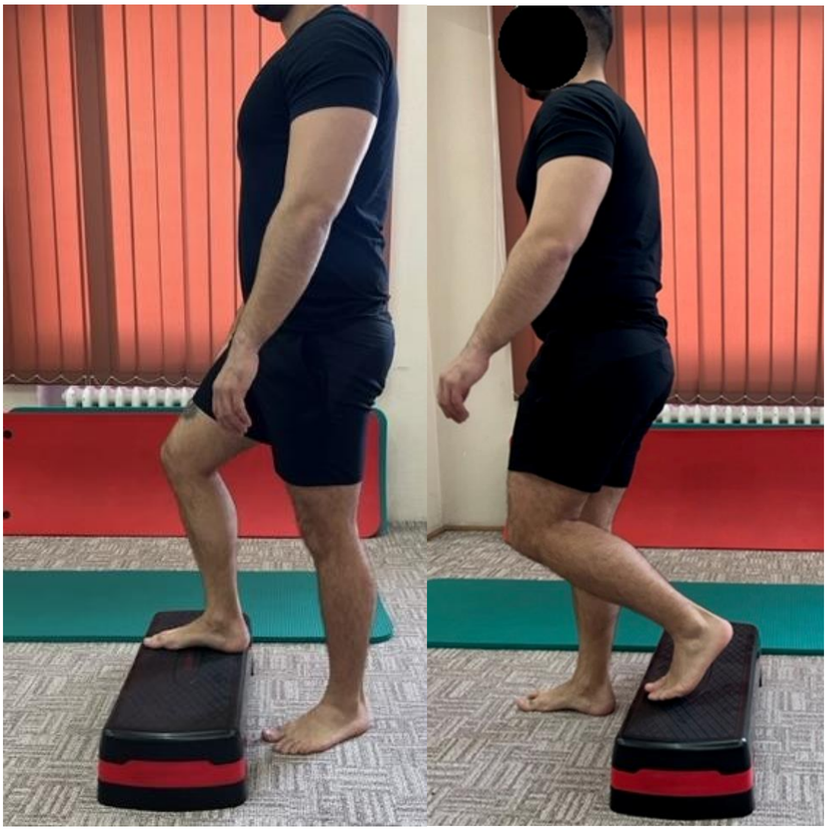

(a) (b)

Figure S5. Subject (a) ascending and (b) descending stairs
